# Study on Spectral Selective Manipulation Characteristics of Surface Multilevel Micro–Nano Structures by FDTD Simulation

**DOI:** 10.3390/ijms23052774

**Published:** 2022-03-02

**Authors:** Xiangjing Guo, Haiying Song, Bairui Du, Shengwang Tan, Shibing Liu

**Affiliations:** 1 Strong-Field and Ultrafast Photonics Lab, Faculty of Materials and Manufacturing, Beijing University of Technology, Beijing 100124, China; gxj1998@emails.bjut.edu.cn (X.G.); dubr123@emails.bjut.edu.cn (B.D.); swtan@foxmail.com (S.T.); sbliu@bjut.edu.cn (S.L.); 2 Key Laboratory of Trans-Scale Laser Manufacturing Technology, Ministry of Education, Beijing University of Technology, Beijing 100124, China

**Keywords:** surface multilevel micro–nano structures, spectral selective manipulation, FDTD

## Abstract

The optical filter based on the micro–nano structure on the material surface is an important optical device, which is widely used in many fields. The filter is fabricated on the substrate with different shapes and sizes of micro–nano array structure, and the wavelength selectivity is realized by adjusting the processing parameters. In this paper, the finite-difference time-domain (FDTD) method is used to simulate the spectral properties of periodic array structures on the Au surface, and the spectral response characteristics of different surface structural parameters to the incident light are obtained. The simulation results show that the periodic pore array has a directional modulation function on the reflectivity and transmittance of the material surface. In the same circular aperture array structure, the wavelength selection ability is proportional to the interval distance of the array period, but the transmission peak linewidth decreases with the increase of the interval distance. The structural spectrum of the cylindrical array is closely related to the structural period. The period of the array structure increases in proportion, the center wavelengths of the reflection and transmission peak of the spectrum are red-shifted. When the height of the array structure increases proportionally, the positions of the center wavelengths of the reflection and transmission peak remain almost unchanged. When the period of the array structure increases, the center wavelength of the reflection and transmission peaks appear red-shifted, and the line width is also narrowed. For the periodic ring array structure, as the inner diameter increases, the reflection peak is significantly red-shifted, and the smaller the ring width, the faster the red-shift of the reflection peak with the wavelength. By controlling the ratio of inner diameter-to-outer diameter, the spectral characteristics of the structured surface can be effectively controlled. These simulation results provide a basis for the preparation of optical filters in the future and a new idea for the study of micro–nano characteristic structures on the surface of materials.

## 1. Introduction

The optical filter is a typical optical device, which can realize the selection of optical wavelength by making the light at a specific wavelength penetrate or cut off [1,2,3,4]. In recent years, with the development of micro–nano processing technology, optical filters based on metal micro–nano structures have been widely used, and have great potential in solar thermal photovoltaic systems, biological sensors, thermal radiators, and other fields [5,6,7]. Especially in the thermal photovoltaic system, an optical filter can regulate the radiation spectrum to achieve optical matching with thermal photovoltaic cells. Moreover, the utilization rate of thermal energy is improved by reflecting mismatch photons, and the working temperature of thermal photovoltaic cells can be reduced [8,9,10,11]. For example, within a layer of Au coaxial circular array, formed by electron beam lithography, is found that the reflection spectrum and transmission spectrum can be effectively adjusted by changing the array period and ring size. The circular hole array structure, with a pore diameter of 150 nm and period of 0.9 μm, prepared on the metal film, is found to enhance the zero-order light of the transmitted light [12]. A system composed of disordered plasma nanoclusters was developed to realize the adjustability of the broadband light response, the broadband absorption of more than 90 of the visible light to the spectral reflection, at a specific optical frequency, was obtained by adjusting the thickness of the external cavity [13]. In addition, the super-transmission spectrum can be effectively controlled by adjusting the geometric shapes of nano-apertures, such as circular, rectangular, and sandwich structures, due to the incident excitation light-generating resonant coupling of surface plasmons at the microstructure array. A series of microstructures constructed are proposed by scientists, such as nanowires, nanoparticles, nano-cones, one-dimensional grating structure, two-dimensional photonic crystals, micro–nano waveguides, which are widely used in the research fields of electronics, microelectronic mechanical systems, and solar photovoltaic devices [14,15,16,17,18,19,20,21,22].

The types of super-surface microstructures are different, so their regulating effects on spectra are also different [23,24,25]. The surface state and bulk state of the material have different optical properties. The symmetry breaking of the surface state leads to the obvious nonlinear optical effect on the surface of the material, which makes the interaction between light and material surface more complex. However, the existence of surface residual bonds and asymmetry of atomic structure make the tension of the linear chain change, resulting in the change of lattice vibration frequency. Therefore, the radiation selectivity regulation from visible light to infrared frequency domain can be realized by designing and preparing micro–nano characteristic structures on the material surface [26]. At present, the mainstream calculation method for the interaction between micro–nano structures and light is the finite difference time domain method (FDTD) [26]. The core idea of FDTD is to use the center difference quotient to realize the first-order deviation of the time and space domains, which can clearly describe the trend of the electromagnetic field in the time domain, show the propagation process of the electromagnetic field, and obtain more simulation information [27,28,29]. In this paper, based on the previous research on the grating structure [30], the spectral simulation of two-dimensional topological characteristic structures, such as the periodic circular hole array, periodic cylindrical array, and periodic ring array structures, is carried out by using the FDTD method. The influence of various physical parameters of the structure on the surface spectrum is simulated, and more periodic topological characteristic structures, with spectral adjustment effects, are explored.

## 2. Simulation Method and Model Design

**Figure 1:**
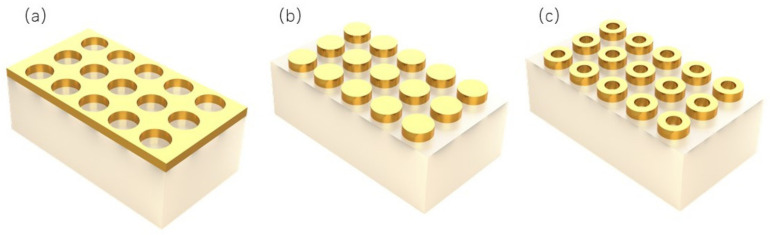
(**a**) Periodic circular hole array, (**b**) periodic cylindrical array, and (**c**) periodic ring array.

As one of the widely used electromagnetic algorithms, the FDTD is one of the indispensable tools for research and product development in the field of optoelectronics. The FDTD is essentially a method for solving Maxwell equations. Its core idea is to discrete the solution space into a rectangular network structure on Cartesian coordinates, where each point is assigned an electric field and a magnetic field, and each point updates the electric field and magnetic field alternately in the form of a frog leaping over time. Au is an important optoelectronic material, and its optical properties are widely used in modern optoelectronic materials and devices. The micro–nano structure of gold has various properties that are superior to those of traditional materials. It covers the wide spectral range from microwave, infrared, and visible ultraviolet, showing the great application prospect of gold-based micro–nano structure in the fields of information and energy. In this paper, three different periodic array structures are established, which are the periodic pore array, periodic cylindrical array, and periodic ring array structures. To avoid the influence of substrate material on the reflection and transmission spectra, the substrate material is set as quartz glass, with a refractive index of 1.45, and the substrate size is set to 2.2 × 2.2 × 1 μm. In the periodic pore array structure, a layer of Au film, with a thickness of 100 nm, is placed on the substrate material, and a series of nano-porous arrays with a diameter of 0.2 μm are etched on the Au film. The period of the nanoporous array is 0.4 μm, as shown in Figure 1a. The incident light source is the linearly polarized plane light in the X-direction, and the wavelength is 0.4–0.85 μm. The X, Y-directions are set to periodic boundary condition, and the Z-direction is set to perfect matching layer (PML) ideal boundary condition. A series of Au cylindrical particles with a radius of 0.1 μm and height of 0.1 μm are placed on the quartz substrate in the periodic cylindrical array structure, and the period between the nano-cylinder arrays is 0.4 μm. The plane wave with a wavelength of 0.4–0.85 μm is selected as an incident light on the material surface, as shown in Figure 1b. Periodic boundary conditions are adopted in X and Y directions, and PML ideal boundary conditions are adopted in the Z-direction. In the periodic ring array structure, an Au ring structure, with an outer diameter of 0.1μm, inner diameter of 0.05 μm, and height of 0.1 μm, is placed above it, and the ring array spacing is 0.4 μm. For example, in Figure 1c, the incident light source is X-direction linearly polarized light, with a wavelength of 0.4–1.35 μm. The X, Y-direction is set to periodic boundary condition, and the Z-direction is PML ideal boundary condition. 

## 3. Simulation Process and Results

### 3.1. Periodic Circular Hole Array Structure

**Figure 2:**
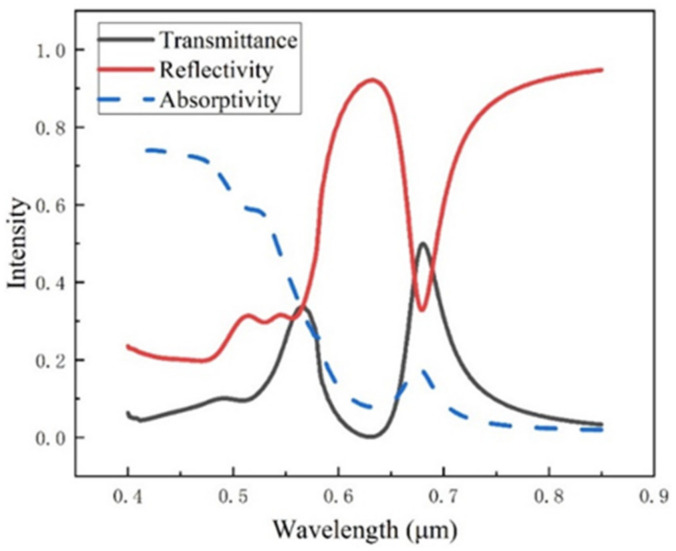
Spectra of periodic circular hole array structure.

Through the optical simulation analysis of the system structure, the transmission spectrum and reflection spectrum of the periodic circular hole array structure are obtained, as shown in Figure 2. It can be seen from the diagram that the reflection spectrum and transmission spectrum show an intense transmission phenomenon at the wavelength of 0.675 μm, corresponding to the valley of the reflection spectrum and peak of the transmission spectrum.

**Figure 3:**
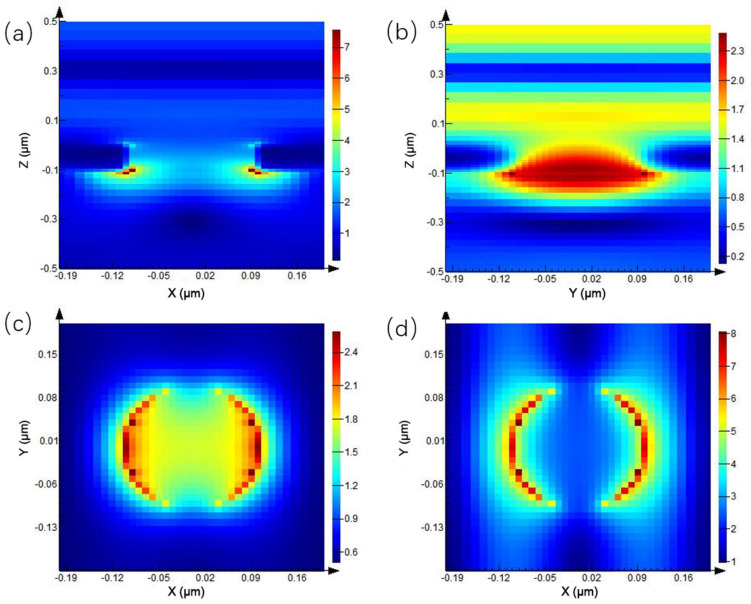
Electric field distribution of periodic circular hole array: (**a**) XZ plane, (**b**) YZ plane, (**c**) above the circular hole array of XY plane, and (**d**) below the circular hole array of XY plane.

Since the transmission peak in the transmission spectrum appears at the wavelength of 0.675 μm, the electric field distribution at different positions at this wavelength is mainly discussed. As shown in Figure 3, Figure 3a is the electric field distribution of the XZ plane of the periodic circular hole array, and the electric field at the junction of the quartz substrate and Au nanohole changes dramatically. The electric field enhancement in the area below the hole is much higher than that in other locations, and the maximum electric field enhancement is seven times. Figure 3b shows the electric field distribution on the YZ plane. Figure 3c,d show the electric field enhancement on both sides of the nanohole, respectively. The local electric field on both sides of the X-axis direction of the nanohole is enhanced, which is due to the use of the X-direction linearly polarized light in the simulation process. Additionally, compared to Figure 3c, the electric field enhancement below the hole in Figure 3d is higher than that above the circular hole array.

**Figure 4:**
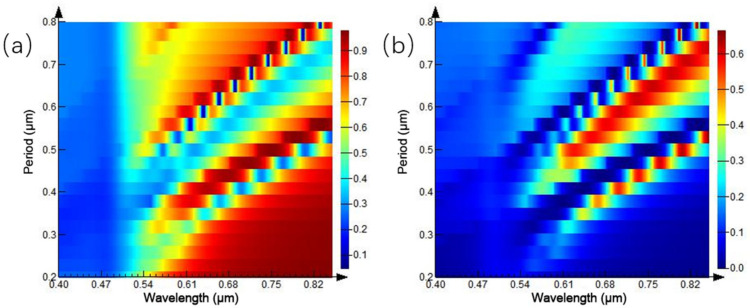
Spectra of equal proportion circular hole arrays with different periods: (**a**) reflection spectrum; (**b**) transmission spectrum.

The original structure parameters are fixed, and the diameter of the circular hole is set to half of the period length, in order to study the influence of the proportion of the nano circular hole array structure on the reflection and transmission spectra under different period conditions. The period of the circular aperture array is set to 0.2–0.8 μm, and the simulation results are shown in Figure 4. Figure 4b is the transmission spectrum of the microstructure array. When the period of the circular hole array is 0.2 μm, a transmission peak appears at the wavelength of 0.54 μm. With the increase of the array period, the intensity of the transmission peak increases progressively and moves red. When the period increases to 0.5 μm, a transmission peak appears again at the wavelength of 0.54 μm, its intensity gradually increases, and the central wavelength of the transmission peak is red-shifted. Figure 4a shows the spectral changes monitored by the reflectivity monitor, and the trend of the reflection spectrum is consistent with the transmittance spectrum.

**Figure 5:**
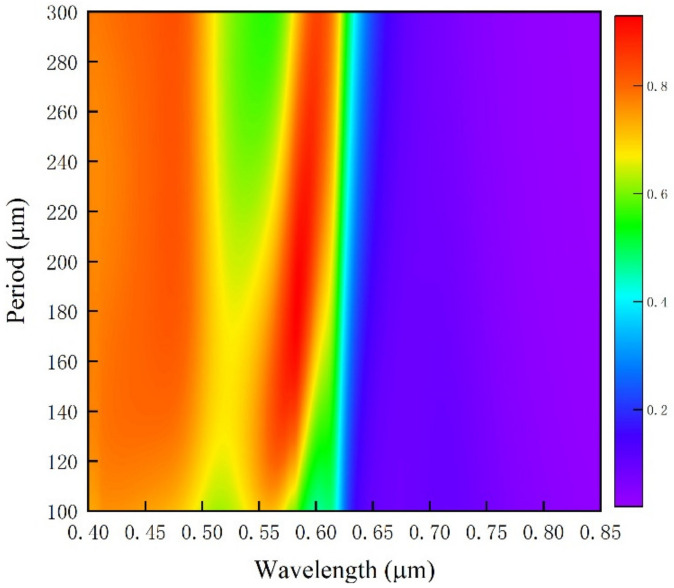
Absorption spectra of equal proportion circular aperture arrays with different periods.

According to the relationship between reflection spectrum and transmission spectrum with a period, the absorption spectrum of periodic circular hole array material is obtained. As shown in Figure 5, with the change of the periodic circular hole array period, the absorption rate of the material changes slightly. The absorption rate near the wavelength of 0.4–0.6 μm is higher, and the absorption rate above 0.65 μm remains low. In summary, periodic hole array direction modulates material surface reflectivity and transmittance.

**Figure 6:**
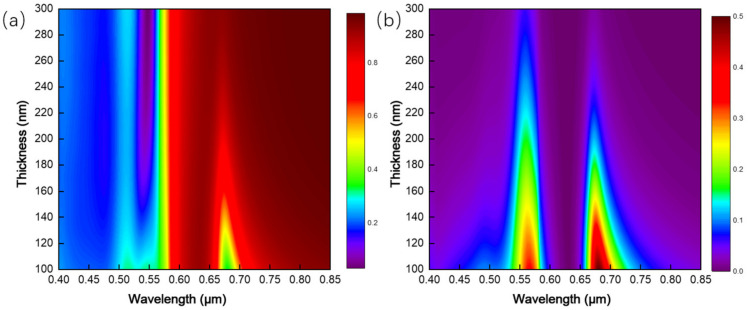
Spectra of periodic circular hole arrays with different thicknesses: (**a**) reflection spectrum; (**b**) transmission spectrum.

Furthermore, the thickness of Au nanofilm is adjusted based on the original simulation structure, and the influence of this parameter change on the spectrum is explored. The thickness of the film is 100–300 nm. Figure 6a,b are the reflection and transmission spectra of the array structure, respectively. The central wavelength of the transmission peak is independent of the thickness of the Au film, and the wavelengths are about 0.69 and 0.56 μm. The intensity of the transmission peak steadily decreases with the increase of Au film thickness. When the Au film thickness reaches 300 nm, the transmission peak disappears. The change of reflectivity spectrum in Figure 6a also proves the changing trend. Therefore, the smaller metal film thickness is better to achieve wavelength selection.

**Figure 7:**
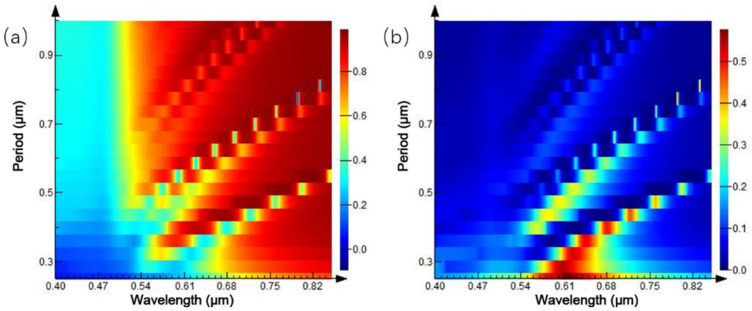
Spectra of circular hole arrays with different intervals: (**a**) reflection spectrum; (**b**) transmission spectrum.

Meanwhile, the period of the circular hole array is simulated to study the influence of periodic hole structure, with different spacing on the reflection and transmission spectra. The circular hole’s radius of the circular hole is 0.1 μm, and the period is 0.25–1 μm. Figure 7b shows the transmission peak in the reflection spectrum. With the gradual increase of the period of the circular hole, the transmission peak redshifts and decreases with the line width. With the continuous increase of the period, the second and third transmission peaks appear gradually and still move to the long band with the increase of the period of the circular hole. The variation trend of the reflectivity spectrum, shown in Figure 7a, is consistent with that of the transmittance spectrum. In the same circular hole structure, the wavelength selection ability is proportional to the spacing distance of the array period, but the transmission peak linewidth decreases with the spacing widening.

### 3.2. Periodic Cylindrical Array Structure

**Figure 8:**
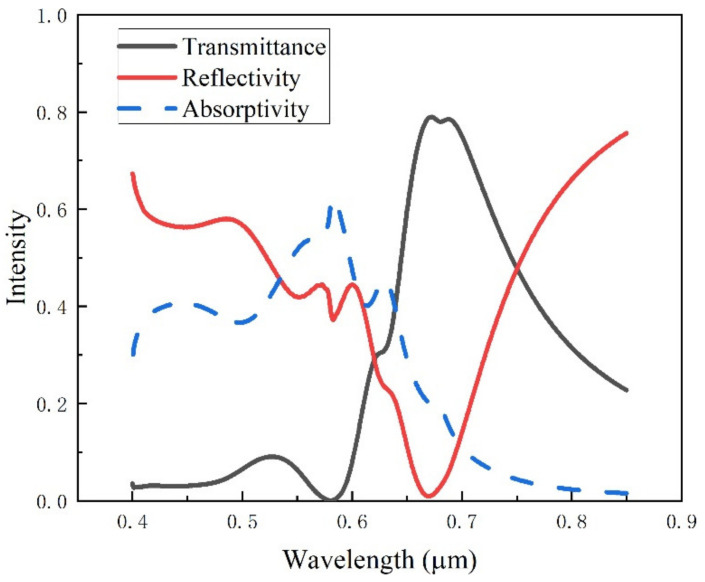
Spectra of periodic cylindrical array structure.

The spectrum simulation of the periodic cylinder structure is shown in Figure 8. A high reflection peak appears at the wavelength of 0.68 μm in the reflection spectrum, and a valley appears at the wavelength in the transmission spectrum. At the same time, the absorption spectrum of the microstructure surface can be calculated according to the reflection and transmission spectra. The absorption spectrum of the periodic cylindrical array structure is consistent with that of the smooth Au film under plane light irradiation.

**Figure 9:**
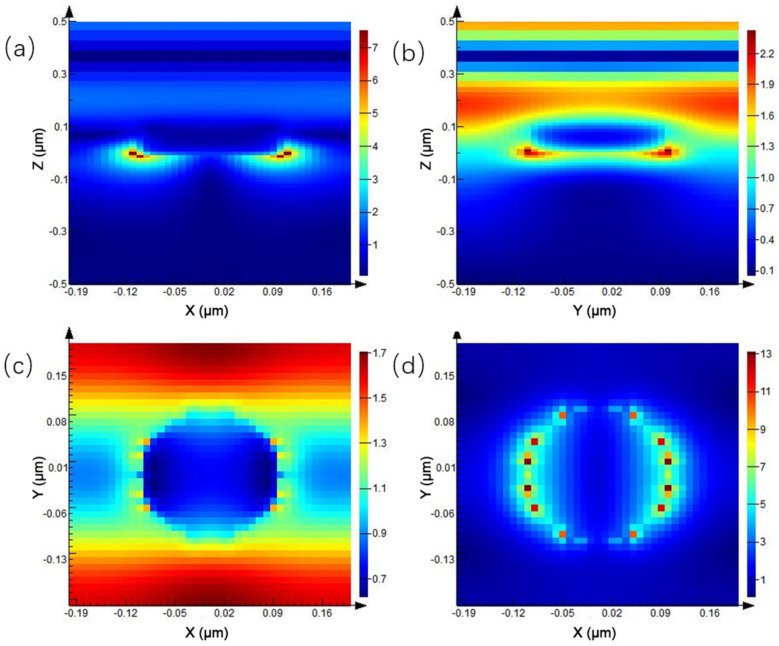
Electric field distribution of periodic cylindrical array: (**a**) XZ plane, (**b**) YZ plane, (**c**) above the cylindrical array of XY plane, and (**d**) below the cylindrical array of XY plane.

Since the reflection peak of the microstructure spectrum is at about 0.67 μm, the electric field distribution of the microstructure, at different positions under this wavelength, is studied. The results are shown in Figure 9, where Figure 9a,b are the distribution maps of the electric field on the XZ plane and YZ plane, respectively.

The electric field enhancement is mainly concentrated below the two ends of Au nano-cylindrical particles, and the maximum electric field enhancement can be achieved by seven times, which is the same as the electric field enhancement of periodic Au nanoholes, under the same parameters. Moreover, the enhancement of the electric field is mainly concentrated in the XZ plane, which is mainly because the electric field of the light source used in the simulation is polarized along the X-axis direction. Therefore, obvious local electric field enhancement can also be observed on both sides of the X-axis direction of the nanoparticles in Figure 9c,d.

**Figure 10:**
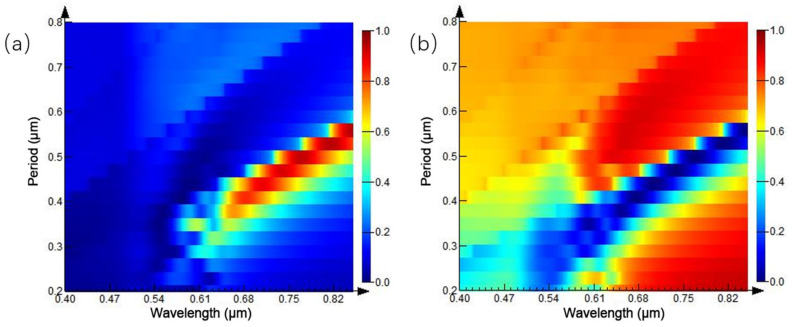
Spectra of equal-proportion cylindrical arrays with different periods: (**a**) reflection spectrum; (**b**) transmission spectrum.

In the simulation process, the structural parameters are fixed, and the cylindrical diameter accounts for half of the proportion of the whole cycle. The spectral changes of the cylindrical array, under different cycles, are studied. The period of the cylindrical array was set to 0.2–0.8 μm, and the results are shown in Figure 10. Figure 10a shows the three-dimensional map of the reflection spectrum under different periodic conditions. During the whole period of variation, the overall reflectivity in the spectral range is low. When the period of the cylindrical array is 0.29 μm, a reflection peak appears at the wavelength of 0.62 μm. The peak intensity of the reflection peak is proportional to the period of the cylindrical array, and the reflection peak redshifts. When the period of cylindrical array reaches 0.6 μm, the reflection peak disappears in the wavelength range of 0.4–0.85 μm. Figure 10b shows the spectrum of transmission spectrum changing with the cylinder period, and the variation trend of the transmission spectrum is the same as that of the reflection spectrum.

**Figure 11:**
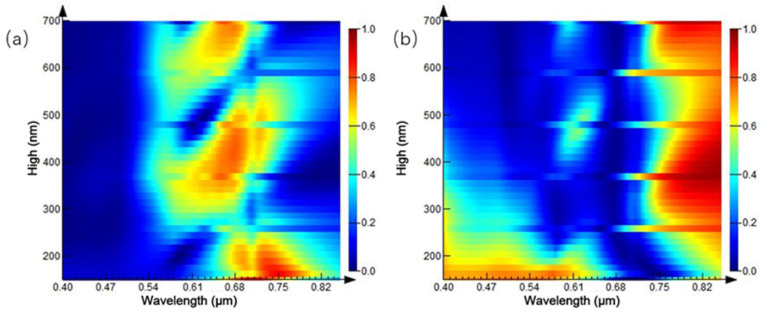
Spectra of equal-proportion cylindrical arrays with different heights: (**a**) reflection spectrum; (**b**) transmission spectrum.

Furthermore, the parameters of the structure are fixed, and the height of the Au nanocylinder array is adjusted based on the initial simulation structure. The influence of the parameter change on the spectrum is simulated. The height of the Au nano-cylinder array is 100–300 nm, and the reflection spectrum is shown in Figure 11a. When the height of Au nano-cylinder array heightens, the reflection peak is evenly distributed near the wavelength of 0.68 μm. When the height of Au nano-cylinder array is 100–250, 300–550, 650–700 nm, a reflection peak appears, respectively, and the linewidth of the reflection peak reaches 100 nm, approximately.

As the height of Au nanoarray augments, the peak shifts to red step-by-step. Figure 11b is the three-dimensional spectrum of the transmission spectrum and height of the Au nanocylinder array. The transmission spectrum is independent of the height and maintains a low transmittance below 700 nm, while the overall transmittance is higher above 0.7 μm. Combined with the spectrum determined by the previous period, it is concluded that the structural spectrum of the cylindrical array is closely related to the structural period, but less related to the height of the array.

**Figure 12:**
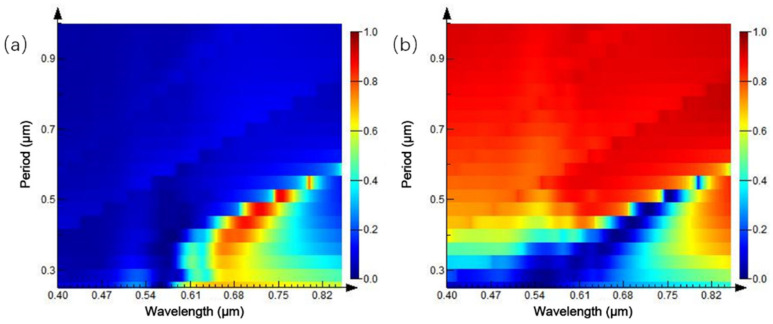
Spectra of cylindrical arrays with different intervals: (**a**) reflection spectrum; (**b**) transmission spectrum.

Finally, the period between the cylindrical array is studied by fixing the original parameters, and the influence of the periodic cylindrical array structure, with different spacing on the reflection and transmission spectra, is researched. The radius of the nanocylinder is 0.1 μm, the height is 100 nm, and the cycle is 0.25–1 μm. In the reflectivity spectrum of Figure 12a, a reflection peak appears between 0.25 and 0.6 μm of the array period. When the period interval becomes larger, the reflection peak redshifts, and the peak intensity strengthens continuously. When the array period is exceeded 0.6 μm, the reflection peak is non-existent. In the wavelength range of 0.4–0.85 μm, the reflection spectrum shows weak reflectivity. Similarly, the same spectral variation trend is observed in the transmission spectrum of Figure 12b. Consequently, the cylindrical array structure, with a large period within a certain range, can achieve an outstanding modulation effect on the reflection and transmission spectra.

### 3.3. Periodic Ring Array Structure

**Figure 13:**
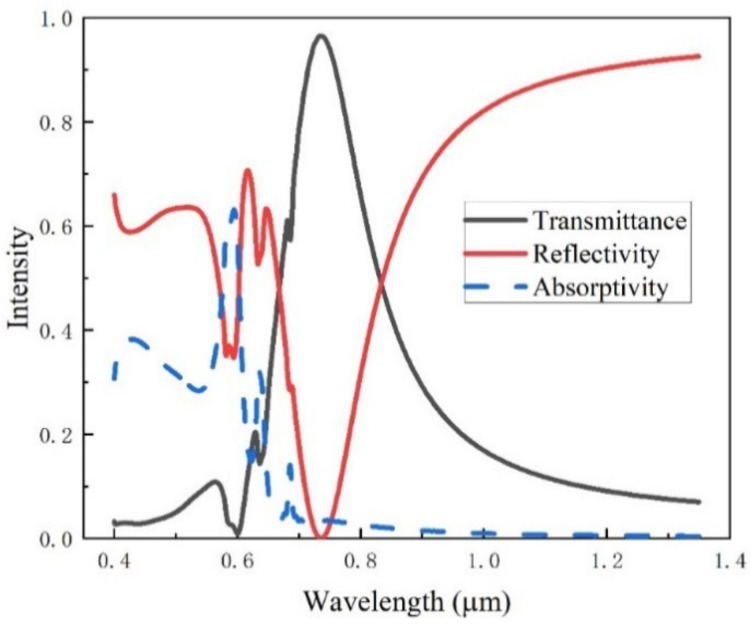
Spectra of periodic ring array structure.

The model is simulated based on the finite difference time domain method. The reflection, absorption, and transmission spectrums of the periodic ring structure are shown in Figure 13. The typical reflection peak appears at the wavelength of 0.73 μm, and the absorption spectrum is the same as that of the smooth Au surface. The absorptivity is high below 0.6 μm, while it is approximately 0 above 0.6 μm.

**Figure 14:**
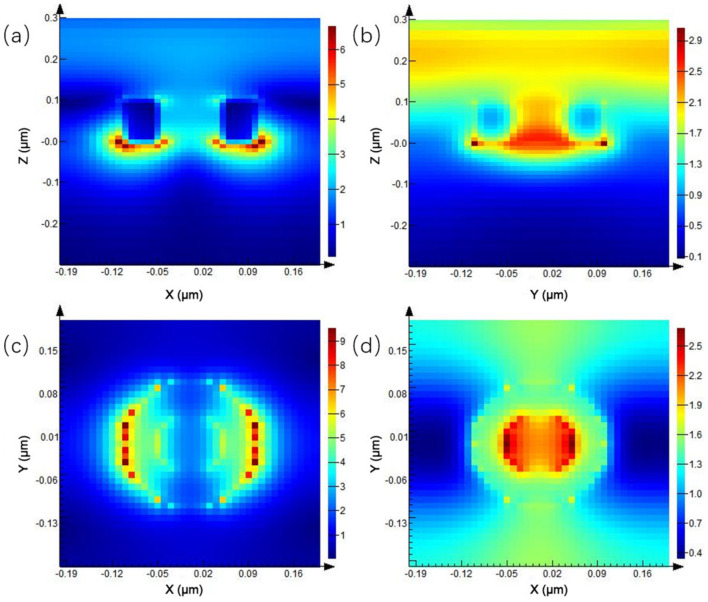
Electric field distribution of periodic ring array: (**a**) XZ plane, (**b**) YZ plane, (**c**) below the ring array of XY plane, and (**d**) above the cylindrical array of XY plane.

Due to the relatively strong electric field change at the wavelength of 0.74 μm, the reflection peak exists in the reflection spectrum at this wavelength. Therefore, the electric field distribution under this wavelength condition was simulated, and the simulation results are shown in Figure 14. Figure 14a shows the electric field enhancement of the XZ plane in the center of the ring. An intense electric field enhancement effect is generated below the Au nanoring array. The electric field enhancement of the outer diameter of the ring is slightly higher than that of the inner diameter, and the maximum electric field enhancement is 6.66 times. Figure 14b is the electric field enhancement of the YZ plane in the center of the ring. It is found that the electric field intensity of the outer diameter is stronger than that of the inner diameter in the Y-axis direction. Because the incident light source is X-axis linearly polarized light, it shows a weak electric field enhancement on the YZ plane.

In Figure 14c, the electric field distribution in the lower transmission direction of the ring shows an intense electric field enhancement on both sides of the X-axis direction of the transmission electric field, and the maximum electric field enhancement is 9.5 times, while the electric field enhancement is not shown in the Y-axis direction. In the electric field distribution, above the ring, in Figure 14d, the electric field enhancement is generally low, and the electric field in the middle of the inner ring is slightly stronger than that outside. It is believed that this is caused by multiple oscillations of electromagnetic waves in the microcavity effect, and the maximum electric field can be enhanced by 2.6 times.

**Figure 15:**
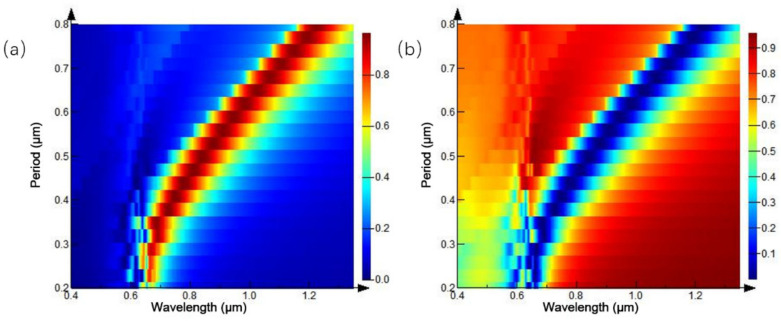
Spectra of equal-proportion ring arrays with different periods: (**a**) reflection spectrum; and (**b**) transmission spectrum.

The ratio of inner diameter:outer diameter:period = 1:2:4 is fixed, and the radius of the ring array is increased to further study the change of reflection and transmission spectra. The simulation results are shown in Figure 15. In the three-dimensional variation of the reflection spectrum in Figure 15a, as the radius of the ring array increases gradually, reflection peak moves to the long-band direction, and maximum reflectivity is close to 1. The transmission spectrum in Figure 15b is basically in accordance. This is consistent with the previous trend of periodic nanoparticles, showing a general rule of nanoarrays.

**Figure 16:**
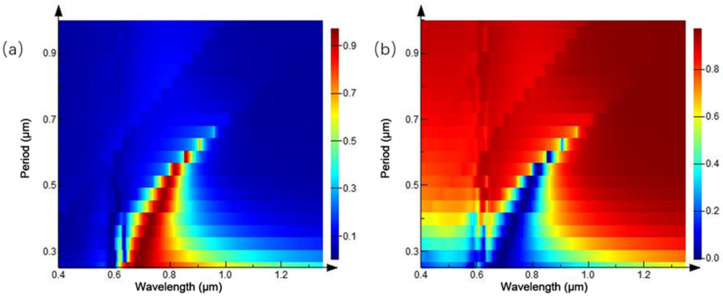
Spectra of ring arrays with different intervals: (**a**) reflection spectrum; (**b**) transmission spectrum.

From the previous analysis, it can be concluded that the reflection and transmission peaks of periodic nanoring array appear red-shifted, with the increase of period, under the condition of constant ratio. So, only changing the period interval of the nanoarrays becomes the focus of the next simulation when the ratio of diameter: outer diameter: period =1:2:4 is no longer maintained. The outer diameter of the fixed Au ring array is 0.1 μm, inner diameter is 0.05 μm, height is 0.1 μm, and period interval is from 0.25 to 1 μm, as shown in Figure 16.

**Figure 17:**
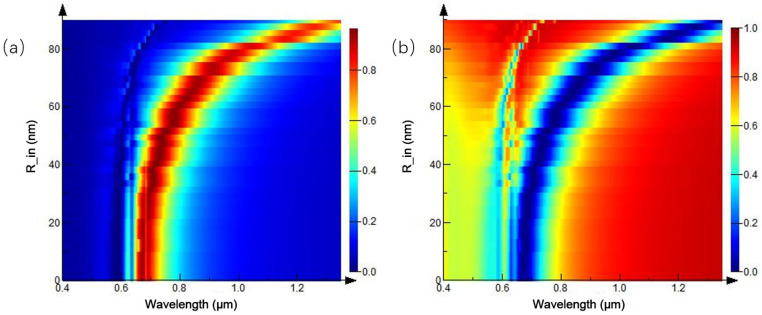
Spectra of ring arrays with different inner diameters: (**a**) reflection spectrum; (**b**) transmission spectrum.

Figure 16a is a three-dimensional variation of the reflection spectrum, with the increase of the period of the Au ring array. When the period interval is 0.25 μm, an asymmetric reflection peak appears at 0.7 μm, and the full width at half maximum (FWHM) of the reflection peak reaches 0.5 μm. With the increase of the array period interval, the central wavelength of the reflection peak gradually moves to the long-wavelength band, and the linewidth of the reflection peak gradually decreases. When the period interval is 0.625 μm, the reflection peak redshifts to the wavelength of 0.91 μm and FWHM has been reduced to 25 nm. According to this, it can be judged that the existence of a peak-like reflection peak can be realized at the period interval of about 0.6 μm. When the period interval is more than 0.66 μm, the reflection peak disappears, reflectivity spectrum generally shows low reflectivity, and relationship with the period interval is weakened. Similar conclusions can be drawn from the variation trend of transmission spectrum in Figure 16b. In consequence, appropriately increasing the periodic interval of the ring array can well-reduce the linewidth of the reflection peak; however, when the interval is super wide, the reflection peak will disappear and incident light wave is fully transparent. The reflection and transmission spectra under different inner diameters are simulated by fixing the outer diameter of Au ring array as 0.1 μm, height as 0.1 μm, and period interval as 0.4 μm. As shown in Figure 16, the reflection spectrum of Figure 16a shows a strong reflection peak at the wavelength of 0.7 μm, when the inner diameter of the ring is 0; that is, the whole array is a nano cylindrical array. With the gradual increase of the inner diameter, the reflection peak moves gradually to the long band at different speeds. When the inner diameter increases from 0 to 33 nm, and the reflection peak’s central wavelength and peak intensity remain stable. When the inner diameter is more than 60 nm, the reflection peak red-shifted rapidly with the increase of the inner diameter, and the smaller the ring width, the stronger the movement rate of the reflection peak. When the inner diameter is 90 nm, the reflection peak is red-shifted to 1.35 μm. It is concluded that the modulation effect of the spectrum can be achieved by controlling the ratio of internal and external diameters, which is consistent with the variation of the transmission spectrum in Figure 17b.

**Figure 18:**
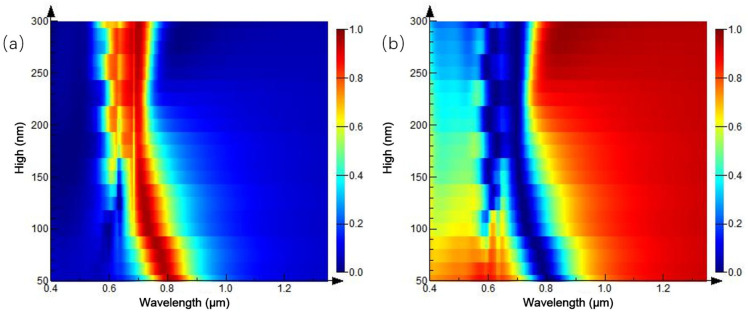
Spectra of ring arrays with different heights: (**a**) reflection spectrum; (**b**) transmission spectrum.

In addition, the outer diameter of the ring array is 0.1 μm, inner diameter is 0.05 μm, and array spacing is 0.4 μm, so that the height of the ring array is controlled in the range of 20–300 nm, and the reflection and transmission spectra are analyzed. The simulation results are shown in Figure 18. In Figure 18a, when the height of the Au ring array is 20 nm, a reflection peak appears at the wavelength of 0.94 μm. With the increase of the height of the Au ring array, the reflection peak gradually blue-shifts and moves to the wavelength of 0.7 μm, at the height of 130 nm. Since then, as the height of the ring array continues to increase, the reflectivity spectrum tends to be stable and no longer moves with the height change, which is consistent with the transmittance spectrum in Figure 18b. Therefore, the wavelength of 0.7 μm is the optimal coupling wavelength of the ring structure, and its position does not change with height. The position of the reflection peak is mainly determined by other parameters of the ring structure.

## 4. Discussion

In the above simulation results, the band absorption lengths of the three different types of surface microstructures on the spectrum are not the same as the regulation effect. In this paper, the surface periodic microstructure designed is equivalent to a series of micro-cavity or micro-cavity-like structures formed on the surface. Based on the surface plasmon polaritons (SPPs) effect [31], that is, the resonant effect of the microcavity is formed on the surface. In the other words, when the microstructure of the material surface (usually referred to as the microcavity structure or the microgroove structure) matches the incident electromagnetic wave in the geometric dimension, the electromagnetic wave travels many times between various surfaces in the cavity and is superimposed in the cavity to form the standing wave. The resonant coupling of the SPPs occurs at the microstructure array of the incident excitation light, and the resonant frequency is determined by the shape and geometric size of the cavity. The existence of the microcavity effect makes many new changes in the photoelectric field on the microstructure surface, which can make some parts of the wavelength light gain and other parts of the light decay. Therefore, the selective control of the light field can be realized by adjusting the microcavity parameters, and the colorimetric purity of the emitted light can be effectively enhanced [32]. The above simulation results found clear support for these above-mentioned views. At the same time, our experimental results also have good verification for previous experimental reports. For example, the researchers found that the light transmittance increased by several orders of magnitude by coupling with the plasma resonance on the surface of the metal film when the light passed through the regularly arranged subwavelength metal holes [33]. By adjusting the geometric shape of nano-apertures, such as circular and rectangular structures, the effective control of the super-transmission spectrum is realized [34,35]. The developed nanoring array Fabry–Perot cavity [36], with double Fano resonance, can well-adjust the resonant wavelength, line width, and wavelength interval of the two Fano resonances. A cross-shaped, two-dimensional cross array structure, designed on the metal surface, realizes the identification of the polarization state of the incident light, while realizing the super-transmission [37]. The resonant regulation of reflection and absorption spectra is realized by controlling the geometric size of the hexagonal silicon nanoarray [38]. The experimental and simulation results show that the designed microcavity or quasi-microcavity structure is very effective for optical filtering, and the light transmission or cut-off, at a specific wavelength, can be realized by different configuration design, that is, the selective regulation of light.

## 5. Conclusions

The traditional method realizes stealth, spectral absorption, and reflection by coating on the surface of the material and sticking to the micro–nano materials. The disadvantage of the traditional method is that long-term exposure leads to the loss of the corresponding function of the material. Therefore, this paper proposed substrate-micro/nano structures integrated characteristic structural materials, and simulated the change of reflection spectral characteristics and selective spectral length caused by different geometric parameters by finite difference time domain method. We use it to simulate three different periodic array structures and obtain the spectral response characteristics of different periodic parameters to the incident light. The results show that as the period of the array structure increases in proportion, the center wavelengths of the reflection and transmission peaks of the spectrum are red-shifted. When the height of the array structure increases proportionally, the positions of the center wavelengths of the reflection and transmission peaks remain almost unchanged. When the period of the array structure increases, the center wavelength of the reflection and transmission peaks appear red-shifted, and the line width is also narrowed. For the periodic ring array structure, as the inner diameter increases, the reflection peak is significantly red-shifted, and the smaller the ring width, the faster the red-shift of the reflection peak with the wavelength. By controlling the ratio of inner diameter-to-outer diameter, the spectral characteristics of the structured surface can be effectively controlled. These simulation results provide a basis for the future experimental development direction of spectral selective regulation and present a new idea for studying the micro–nano characteristic structure of the material surface. The multi-level and multi-scale micro/nano- characteristics structures will be fabricated on the material surface, to form an equivalent harmonic oscillator of more adaptive intrinsic frequencies, and try to develop the control technology of the radiation spectrum with broadband domain selectivity from visible light to infrared on dielectric and metal materials. Finally, we wish the achieved mechanism and the technology can help the micro-devices such as micro-aircraft or submerge vehicles to be of high durable transform spectrum, surface radiation light or heat, etc., in the engineering applications and act as a complementary application technology for the metamaterials fabrication.

## Data Availability

Date can be available upon request from the authors.
